# A comparative study on point cloud down-sampling strategies for deep learning-based crop organ segmentation

**DOI:** 10.1186/s13007-023-01099-7

**Published:** 2023-11-11

**Authors:** Dawei Li, Yongchang Wei, Rongsheng Zhu

**Affiliations:** 1 Engineering Research Center of Digitized Textile and Fashion Technology, Ministry of Education, Donghua University, Shanghai, 201620 China; 2 College of Information Sciences and Technology, Donghua University, Shanghai, 201620 China; 3 State Key Laboratory for Modification of Chemical Fibers and Polymer Materials, College of Information Sciences and Technology, Donghua University, Shanghai, 201620 China; 4 College of Arts and Sciences, Northeast Agricultural University, Harbin, 150030 China; 5 National Key Laboratory of Smart Farm Technology and System, Harbin, 150030 China

**Keywords:** Plant phenotyping, Point cloud down-sampling, Deep learning, Crop organ segmentation, 3D crop data

## Abstract

The 3D crop data obtained during cultivation is of great significance to screening excellent varieties in modern breeding and improvement on crop yield. With the rapid development of deep learning, researchers have been making innovations in aspects of both data preparation and deep network design for segmenting plant organs from 3D data. Training of the deep learning network requires the input point cloud to have a fixed scale, which means all point clouds in the batch should have similar scale and contain the same number of points. A good down-sampling strategy can reduce the impact of noise and meanwhile preserve the most important 3D spatial structures. As far as we know, this work is the first comprehensive study of the relationship between multiple down-sampling strategies and the performances of popular networks for plant point clouds. Five down-sampling strategies (including FPS, RS, UVS, VFPS, and 3DEPS) are cross evaluated on five different segmentation networks (including PointNet +  + , DGCNN, PlantNet, ASIS, and PSegNet). The overall experimental results show that currently there is no strict golden rule on fixing down-sampling strategy for a specific mainstream crop deep learning network, and the optimal down-sampling strategy may vary on different networks. However, some general experience for choosing an appropriate sampling method for a specific network can still be summarized from the qualitative and quantitative experiments. First, 3DEPS and UVS are easy to generate better results on semantic segmentation networks. Second, the voxel-based down-sampling strategies may be more suitable for complex dual-function networks. Third, at 4096-point resolution, 3DEPS usually has only a small margin compared with the best down-sampling strategy at most cases, which means 3DEPS may be the most stable strategy across all compared. This study not only helps to further improve the accuracy of point cloud deep learning networks for crop organ segmentation, but also gives clue to the alignment of down-sampling strategies and a specific network.

## Introduction

Crops are very important to human beings. Throughout the human history, crops have been playing an important role in both people’s livelihood and social development. Crops are indispensable to food, agriculture industry, husbandry, environmental protection, energy [1–4], and other related aspects. Observation of the changes upon crop phenotypes during cultivation is of great significance to screening excellent varieties and improving crop yield. The crop phenotypes refer to a class of measurable characteristics and external traits of crops. Phenotypes are the result of the interaction between the intrinsic gene expression and the external environmental influences on crops, and are determined as an important factor cluster that determines yield, quality, and stress resistance [5]. Specifically, the crop phenotypes include the structure of the crop, the shape and density of the stem and leaf, and the process of growth and development [6]. The modern tools and sensors have greatly facilitated various crop phenotyping applications, especially those focusing on automatic feature calculation. The first and the key task in phenotyping is to identify and segment all organ instances of crops, so that the automatic calculation that follows can work correctly. Therefore, automatic organ segmentation based on different data forms has becoming a mainstream direction of the crop phenotyping research.

Since the beginning of this century, plenty of research on plant (including organ) segmentation based on two-dimensional images has been published, such as methods based on thresholding [7–12], edge detection [13–16], region growing [17–19], clustering [20–25] and deep learning [26–33]. Although remarkable progress has been made in the field of crop image phenotyping, 2D images are intrinsically the projections of 3D shapes, which inevitably results in information loss. Therefore, recent phenotyping research based on 3D crop data has become a new direction. The most widely used form of 3D data is the point cloud, and its acquisition measures can be roughly divided into structured light systems, indirect Time-of-Flight (iToF) cameras, high-precision direct ToF (dToF) sensors (LiDAR), and stereo vision/multi-view stereo (MVS) [34]. The 3D point cloud data of plants acquired by the LiDAR has been widely used in 3D reconstruction and phenotyping of trees, maize, cotton, and other crops [35–42]. Although LiDAR has high precision, its data acquisition cost is high. Kinect Azure (Kinect V3) [43] and commercial iToF sensors take into account both cost and speed, and can quickly obtain 3D point clouds of crops at the expense of losing certain accuracy. The binocular stereo sensors such as ZED [44] can be used for 3D phenotyping tasks. The compact size of stereo sensors has made them to be easily applied on robots and UAVs, facilitating rapid and high-throughput plant phenotyping. The Intel RealSense D415/D435 series [45], which are based on the infrared structured light, can be used to obtain depth images and coarse point clouds of large crops in real time.

Accurate segmentation of plant organs on reliable 3D plant point cloud data is both the focus and the difficulty of 3D crop phenotyping. With the breakthrough of machine learning and artificial intelligence in recent years, deep learning methods for unordered and unevenly distributed data such as 3D point clouds have made great progress in performance. For point cloud semantic segmentation tasks, early deep learning-based models cannot work directly on point clouds; they rely on multi-view representation, which usually first projects a point cloud onto 2D images and applies image-based deep neural networks to segment, and then conducts back-projection to map 2D results back into 3D space. Some representative studies include Multi-View [46–48] and Spherical Images [49, 50], followed by 2D CNN segmentation. The main drawback of methods of this kind is that the geometrical 3D data is not fully exploited, and the projection and back-projection processes inevitably lose some details. In order to reduce information loss, several studies switch to voxelization [51–57], which replaces the original point cloud data with a number of voxels, and then carries out 3D convolution on the grid to extract deep crop features and perform organ segmentation. However, the computation complexity of 3D convolution is high, and voxelization strongly smooths the point cloud distribution, dropping some local geometrical information. Therefore, since recently, direct deep learning on points has become a key research direction. Qi et al. [58] proposed a pioneering network PointNet that used shared Multi-layer Perceptron (MLP) to learn point-level features, and utilized max-pooling layer to extract the global features. PointNet realized end-to-end crop point cloud classification and semantic segmentation tasks at the point-level. PointNet +  + [59] used the encoding–decoding framework to improve the local feature learning of PointNet. Much research since then has been dedicated to improving the computational framework of the PointNet family, and efforts are being made on modification of those networks to adapt the plant phenotyping tasks.

To improve the weakness of PointNet +  + that often focuses on sole point-level features but ignores the point-point connections, Wang et al. [60] designed Dynamic Graph Convolutional Neural Network (DGCNN) for integrating the relationship between points into point cloud processing, and proposed a dynamically updated graph convolution block called EdgeConv. Li et al. [61] designed a dual-function point cloud deep learning network PlantNet, which uses a dual-pathway architecture to achieve semantic segmentation and instance segmentation at the same time. PlantNet achieved better plant organ segmentation results than PointNet +  + , SGPN [62], and ASIS [63] on a comprehensive crop dataset. Ghahremani et al. [64] proposed Pattern-Net to segment wheat point clouds. Pattern-Net used KNN to aggregate different features to make the network more robust to changes in point cloud density, distortion, and noise level. Gong et al. [65] designed a 3D point cloud convolutional neural network based on PointConv [66] module to effectively segment panicles for rice point clouds. Li et al. [67] designed a deep learning network PSegNet that can be applied to multiple types of crop point clouds to achieve semantic and instance segmentation simultaneously. In the network architecture, PSegNet contains a dual-granularity feature fusion module, a mixture of the attention modules [68] that helps to achieve satisfactory segmentation performance.

High-quality plant point cloud data usually have problems such as huge number of points, uneven density, and frequent occurrence of outliers; therefore down-sampling is usually required for data preprocessing and compression. In addition, the training of the deep learning network requires the input point cloud to have a fixed scale. All point clouds in the training batch should have similar scale and contain the same number of points, which puts forward a high requirement for down-sampling of point clouds. Choosing an appropriate down-sampling method can not only reduce the impact of noise, but can also preserve the most important 3D spatial structure as much as possible. At present, there are several popular strategies for down-sampling of point clouds. The Farthest Point Sampling (FPS) [69] is perhaps the most commonly used down-sampling method for point clouds. It can ensure that the sampled points have global coverage and the number of points can be fixed. But, the FPS requires to traverse a distance calculation from each point to the rest of all points, so that the computational complexity approaches *O*(*n*^2^) in implementation (n to be the number of points). Random Sampling (RS) [70] is a sequential random sampling method. It has the advantages of low calculation complexity (can be as low as $O(n)$) and fast speed in implementation. It can also strictly control the number of down-sampling points. However, it may deteriorate the non-uniformity in point cloud density, i.e., the sparse area becomes even sparser compared to other regions after sampling. The voxel-based sampling first defines a three-dimensional grid on the point cloud, and then selects a point to replace all points in the voxel to achieve the goal of reducing the complexity of the point cloud. The replacement point in the voxel can be chosen by either the gravity centroid of the voxel body or the original point that is closest to the centroid. The two corresponding voxelized down-sampling strategies are called Uniformly Voxelized Sampling (UVS) [71] and Voxelized Farthest Point Sampling (VFPS) [67], respectively. 3D Edge-Preserving Sampling (3DEPS) [61] draws inspiration from human sketching. In 3DEPS, the 3D Surface Boundary Filter [72] is first applied to divide point cloud into two parts, edge points and internal points, and the two parts are combined into a new point cloud by artificially adjusting the proportion of the edge points. 3DEPS believes that by introducing adequately more edge points during down-sampling can improve the training and segmentation performance of point cloud segmentation networks.

At present, most deep networks for 3D phenotyping only tried a single down-sampling algorithm such as FPS to prepare training sets and test sets. There is a lack of comprehensive evaluation on down-sampling strategies for point cloud deep networks in the overall research field. The adaptability between the sampling measures and those deep networks is still unclear. As far as we know, this paper is the first comprehensive study of the relationship between multiple down-sampling strategies and the performances of popular networks for plant point clouds. This work not only helps to further improve the accuracy of point cloud deep learning networks for crop organ segmentation, but also gives clue to answering the question of what kind of down-sampling strategy should be applied on a specific network. In addition, this study may also shed new light on designing new down-sampling algorithms for unordered data. The main contributions of this paper are as follows:

(i) This paper first explores the feasibility of several down-sampling strategies to generate crop point cloud datasets for deep learning, and successfully forms crop point cloud datasets (containing three species) under five different down-sampling strategies with a fixed number of points, respectively. These five down-sampling strategies are Farthest Point Sampling (FPS) [69], Random-Sampling (RS) [70], Uniformly Voxelized Sampling (UVS) [71], Voxelized Farthest Point Sampling (VFPS) [67], and 3D Edge-Preserving Sampling (3DEPS) [61].

(ii) The five down-sampling strategies (including FPS, RS, UVS, VFPS, and 3DEPS) are cross evaluated on five mainstream point—level deep networks (including PointNet +  + [59], DGCNN [60], PlantNet [61], ASIS [63], and PSegNet [67]) for plant organ segmentation. The overall experimental results show that currently there is no strict golden rule on selecting down-sampling strategy on mainstream crop deep learning networks, and also reveal that the optimal down-sampling strategy may vary among different networks.

(iii) Though the current experiments strongly prove the non-existence of a “golden” down-sapling strategy, several broad and relaxed clues can be summarized for selection of suitable sampling strategies. First, 3DEPS and UVS tend to generate better results on semantic segmentation networks. Second, the voxel-based down-sampling strategies may be more suitable for complex dual-function networks. Third, at 4096-point resolution, 3DEPS usually has only a small margin compared with the best down-sampling strategy at most cases, which means 3DEPS may be the most stable strategy that obtains sub-optimal results across all compared.

The acronyms and notations used in this paper are summarized in Table 1. The rest of the paper is arranged as follows. Methods of down-sampling and the networks that will be tested in this paper are explained in "Methods" section. The datasets and details in experimental configuration are elaborated in "Experiments" section. The quantitative and qualitative results are given in "Results" section, together with summary and suggestions. Discussion on a high-precision plant dataset is provided in "Discussion" section. Finally, the conclusion is drawn in the last section.

**Table 1:** Acronyms and notations

FPS	Farthest Point Sampling
3DEPS	3D Edge-preserving Sampling
RS	Random Sampling
UVS	Uniformly Voxelized Sampling
VFPS	Voxelized Farthest Point Sampling
VBS	Voxel-based Sampling
SBF	3D Surface Boundary Filter
LFEOs	Local Feature Extraction Operations
FFM	Feature Fusion Module
LFEM	Local Feature Extraction Module
DGFFM	Dual-granularity Feature Fusion Module
GT	Ground truth
TP	True Positive
FP	False Positive
FN	False Negative
IoU	Intersection over Union
AveDiff	The average difference to the best performer
mCov	Mean coverage
mWCov	Mean weighted coverage
${\mathcal{P}}$	The original point set or point cloud
$M$	The number of points in the original ${\mathcal{P}}$
$n$	The number of points after down-sampling
$m$	The number of remaining points to be sampled
$N$	Number of points not yet visited in ${\mathcal{P}}$
$S(m,N)$	The random variable used in Random Sampling
$F( \cdot )$	A probability distribution function
$A:$	Permutation
$U,V$	Random variables with a uniform distribution in (0, 1)
$A \leftarrow B$	Assign the value B to A
${\mathcal{P}}_{i}$	The points contained in the i-th voxel
$c_{i}$	The gravity centroid in XYZ space of ${\mathcal{P}}_{i}$
$l_{x}$, $l_{y}$,$l_{z}$	The length, width, and height of each voxel
${\mathcal{C}}$	Internal point set
${\mathcal{B}}$	Edge point set
$C$	The number of semantic classes
$C_{ins}$	The number of semantic classes that have instances
*IoU* $( \cdot, \cdot)$	Intersection over Union calculation of two entities

## Methods

This section mainly explains the methodology of our study. Sub-Section "Down-sampling strategies" will revisit the five popular down-sampling strategies evaluated in the study, including the general description of the implementation as well as the speed and the characteristics of each strategy. Sub-Section "Deep networks for plant point clouds" will review the five deep learning networks tested for plant organ segmentation. Among the five networks, PointNet +  + and DGCNN are single-function segmentation networks, and can only realize organ semantic segmentation in crop point clouds. The other three networks—ASIS, PlantNet, and PSegNet realize organ semantic segmentation and leaf instance segmentation at the same time.

### Down-sampling strategies

**Fig. 1:**
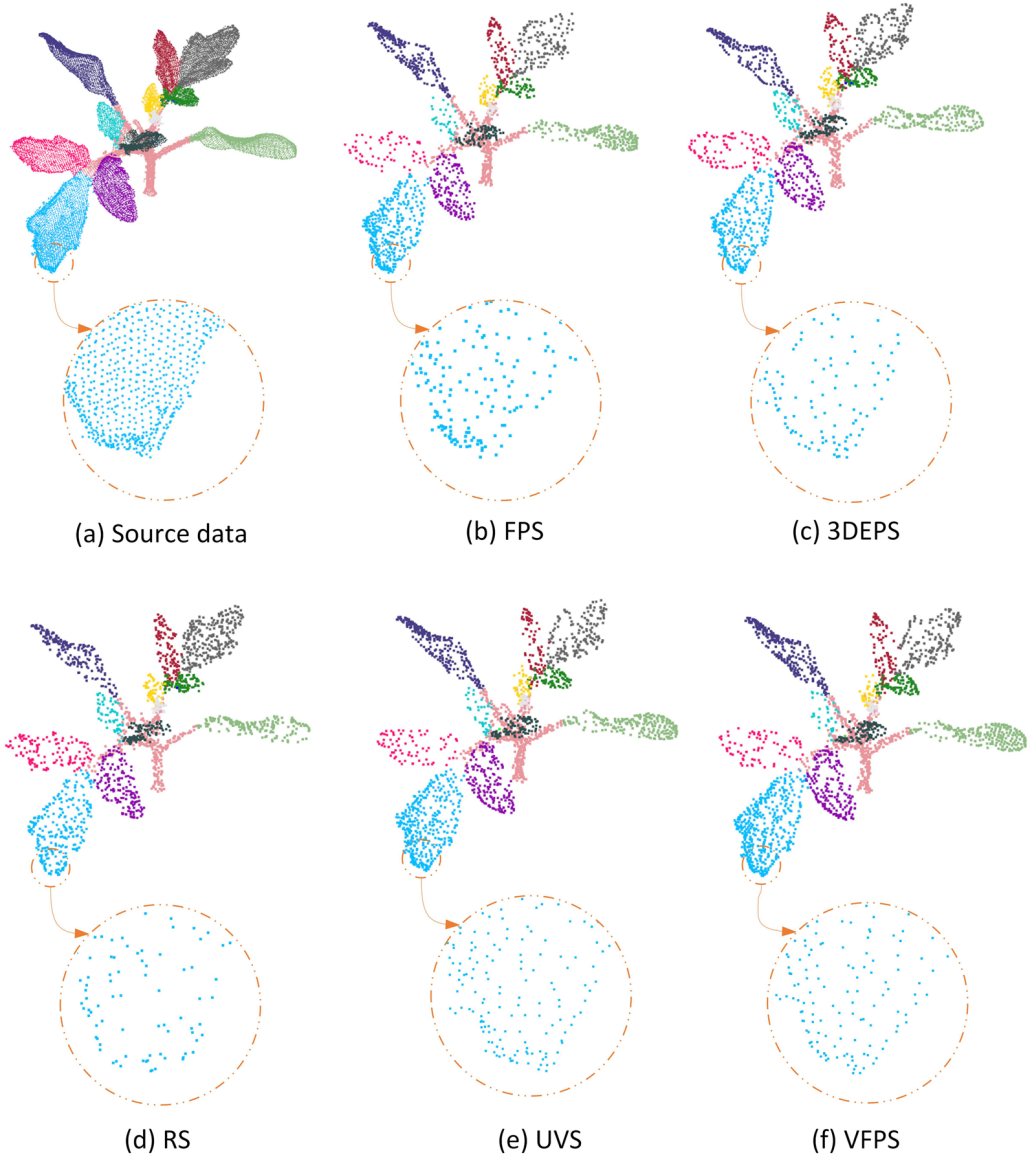
Qualitative demonstrations of the five down-sampling strategies studied in this paper. **a** An original tomato plant point cloud with human-labeled leaf instances and stem system, containing a total of 18,521 points. **b** Down-sampled point cloud with 2,000 points using FPS. **c** Down-sampled point cloud with 2000 points using 3DEPS (with a ratio of 0.2). **d** Down-sampled point cloud with 2000 points using RS. (e) Down-sampled point cloud with 2,000 points using UVS. **e** Down-sampled point cloud with 2,000 points using VFPS

Theoretically, the point-level deep learning network can accept an input of any size. But an excessively large number of input points will lead to an abrupt increase in network parameters; hence, this will significantly slow down the training speed. In addition, redundant input points have little effect on improving the training results and can even cause overfitting. Therefore, down-sampling of point clouds is essential for current point-level deep learning framework. In this sub-section, we will mainly revisit the principles of the five down-sampling strategies (FPS, RS, UVS, VFPS, and 3DEPS) and their performances on crop point clouds. Figure 1 shows visualizations of five down-sampling strategies on a dense tomato plant point cloud, respectively.

#### Farthest Point Sampling (FPS)

The Farthest Point Sampling (FPS) strategy [69] repetitively selects the farthest point to perform down-sampling. First, it randomly selects a $p_{0}$ point from the original point cloud ${\mathcal{P}}$ as the starting point, pushes this point into the point set ${\mathcal{A}}$. Second, each time it traverses the point set ${\mathcal{P}\backslash \mathcal{A}}$ to compute the sum of the distances from all points in ${\mathcal{A}}$ to all points in ${\mathcal{P}\backslash \mathcal{A}}$. After locating the point $p$ that has the minimum sum of distances, then remove $p$ from ${\mathcal{P}\backslash \mathcal{A}}$ and put it into ${\mathcal{A}}$, and do over this process until the number of points in point set ${\mathcal{A}}$ satisfies the requirement. The algorithm has a complexity of $O(Mn)$, where $n$ is the number of points after down-sampling, and $M$ is the number of points in the original point cloud ${\mathcal{P}}$. FPS is widely used because of its simple implementation and relatively uniform sampling effect. The disadvantage is that after down-sampling, it is easy to deteriorate the non-uniformity in point distribution, and when the number of $n$ is far less than $M$, FPS tends to have holes inside objects. The FPS down-sampling result of a tomato point cloud (Fig. 1a) is visualized by Fig. 1b.

#### Random sampling (RS)

Random sampling (RS), which is generally implemented in sequentially random sampling algorithms [70], has a time complexity of $O(M)$. The algorithm first defines a random variable $S(m,N)$, where $m$ is the number of remaining points to be sampled by RS, and $N$ is the number of points that have not been traversed in the original point cloud ${\mathcal{P}}$. This random variable $S$ represents the number of points to be skipped before sequentially selecting the next point in the point cloud, the sequential scan can be regarded as traveling only once and one way on the point cloud sequence. As $n$ represents the number of points to be sampled, then $n - m$ represents points that have already been sampled, the next point index calculated by RS is the $(S(m,N) + 1)th$ point after the current search position in the original point cloud sequence. The probability distribution function of $s$ can be defined by (1).

1 $$F(s) = P(S \le s) = 1 - \frac{{A_{N - s - 1}^{m} }}{{A_{N}^{m} }} = 1 - \frac{{A_{N - m}^{s + 1} }}{{A_{N}^{s + 1} }}{\text{ with }}0 \le s \le N - m.$$

In order to find a suitable and smooth $S(m,N)$, an uniform random variable $U$ between 0–1 can be used to make $U \le F(s)$. Considering Eq. (1), the variable $U$ obeys the inequality (2):

2 $$U \le 1 - \frac{{A_{N - m}^{s + 1} }}{{A_{N}^{s + 1} }}.$$

Let $V = 1 - U$, and because $U$ is a random variable with a uniform distribution of 0–1, $V$ is also a random variable obeying a uniform distribution of interval (0, 1). After rearranging inequality (2), we obtain

3 $$A_{N - m}^{s + 1} \le A_{N}^{s + 1} V.$$

In the actual implementation, the integer variable $s$ is cycled from 0 each time, and the random variable $V$ is regenerated in each cycle, and it is then tested if it satisfies Eq. (3). If not satisfied, $s$ is incremented by 1 until satisfied. If Eq. (3) is satisfied, the cycle then quits and at this time we let $S(m,N) = s$, the $(S(m,N) + 1)th$ point is sampled. In the next round of calculation, let $N \leftarrow N - S(m,N) - 1$ and $m \leftarrow m - 1$. At the same time, we let $s \leftarrow 0$. The sampling ends until $m = 0$.

The RS down-sampling method is the fastest across all the strategies investigated in this paper. Its performance relies heavily on the density distribution of the original data structure. The RS down-sampling result of a tomato point cloud (Fig. 1a) is visualized by Fig. 1d.

#### UVS and VFPS

Voxel-based sampling (VBS) is to construct voxels in the three-dimensional space of the point cloud. The length, width, and height of each voxel are defined by $l_{x}$, $l_{y}$, and $l_{z}$, respectively, and act as input parameters. Then select a selects a point to replace all points in the voxel to achieve the goal of reducing the complexity of the point cloud. In this paper, we focus on two different VBS strategies on selecting the replacement point in each voxel: Uniformly Voxelized Sampling (UVS) [71] and Voxelized Farthest Point Sampling (VFPS) [67].

Taking the *i-th* voxel as an example, the points contained in the voxel form a set ${\mathcal{P}}_{i}$. The Uniform Voxel Sampling (UVS) [71] replaces each cube (voxel) with the real point that is closest to the geometric center of the cube in ${\mathcal{P}}_{i}$, and then filters the total number of sampled points to the set value with FPS. The Voxelized Farthest Points Sampling (VFPS) [67] replaces each cube with the gravity centroid $c_{i}$ of the set ${\mathcal{P}}_{i}$, and then uses FPS strategy to fix the number of sampled points. The speed of voxelized sampling strategies are fast, because it can effectively reduce the complexity of the point cloud while maintaining the global shape and smoothing the holes in point clouds. However, three disadvantages still exist: (i) the three parameters for voxelization $l_{x}$, $l_{y}$, $l_{z}$ need to be manually adjusted according to the characteristics and distribution of different sources of point clouds; (ii) the number of points after the voxelization operation is uncertain, and needs an extra FPS step to fix the number of points later, which increases algorithm complexity; and (iii) once the size of the voxel is determined, the structure of all point clouds after the down-sampling are basically similar in density, which may cause overfitting during training. The UVS and VFPS down-sampling results of a tomato point cloud (Fig. 1a) are visualized by Fig. 1e and Fig. 1f, respectively.

#### 3D Edge-Preserving Sampling (3DEPS)

3D Edge-Preserving Sampling (3DEPS) [61] imitates the shape abstraction method of sketching, and effectively describes complex 3D objects by outlining the sharp edges of objects under limited resources. 3DEPS first uses the 3D Surface Boundary Filter (SBF) [61] to divide the point cloud organ into two parts: edge points and internal points, and then adjusts the ratio of the two parts to “re-build” a new point cloud. In general, more edge points can be artificially introduced to make the restructured point cloud retain more edge information. Specifically, the original point cloud ${\mathcal{P}}$ is first divided into edge point set ${\mathcal{B}}$ and internal point set ${\mathcal{C}}$ by SBF, and then FPS is applied to point set ${\mathcal{B}}$ and point set ${\mathcal{C}}$ respectively according to the ratio parameter set beforehand. Finally, the two parts of points are combined to form the final point cloud with an exact number of points. 3DEPS has two obvious advantages: (i) it can artificially adjust the ratio of edge points and internal points according to user’s need, and (ii) the introduction of FPS that follows not only control the exact number of final sampled points, but also bring a certain randomness to 3DEPS, making it easier to perform data enhancement for the training of deep networks. The disadvantage is that the steps of the strategy are more complicated than those of FPS and RS, and the ratio of the edge points to the total number of points is a parameter to be tuned experimentally. The 3DEPS down-sampling result of a tomato point cloud (Fig. 1a) is visualized by Fig. 1c.

### Deep networks for plant point clouds

The application of deep learning on point cloud data has produced fruitful results, maintaining an evident edge in tasks such as classification, semantic segmentation, and instance segmentation over non-deep methods. In recent years, generic point cloud deep networks PointNet +  + [59], DGCNN [60], and ASIS [63] have achieved satisfactory accuracy on CAD point cloud models such as ShapeNet [73]. At the same time, some networks specially designed for plant point cloud data have also emerged, e.g., PlantNet [61] and PSegNet [67]. They have strong variety adaptability and can realize semantic segmentation and instance segmentation tasks simultaneously. In this sub-section, we will briefly introduce the basic frameworks of five popular deep learning in the field of crop phenotyping, respectively.

**Fig. 2:**
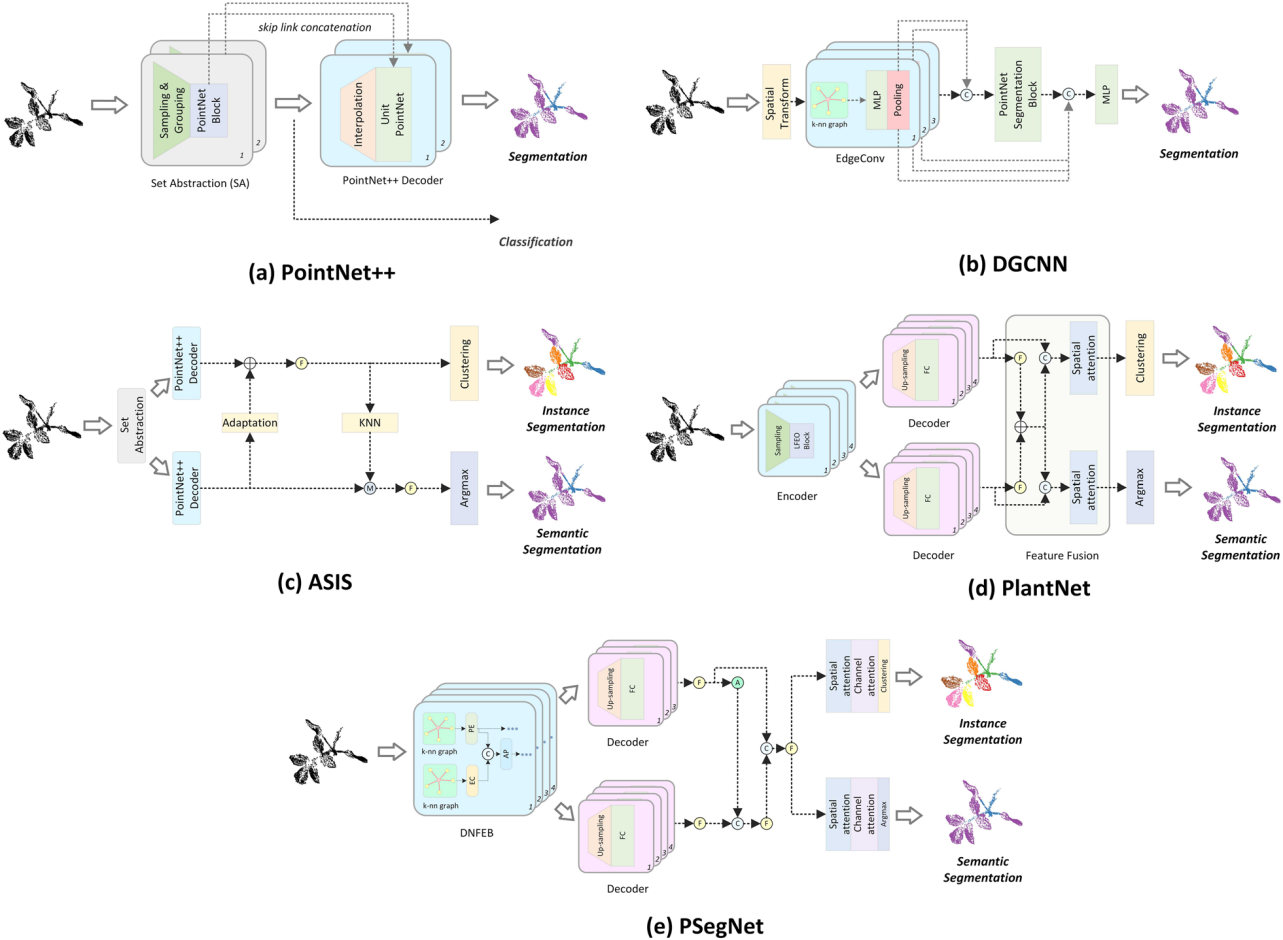
The structure or pipeline of the five networks evaluated in this paper. **a** shows the architecture of PointNet +  + ; **b** shows the architecture of DGCNN; **c** shows the architecture of ASIS; **d** shows the architecture of PlantNet; **e** shows the architecture of PSegNet

PointNet +  + [59] is a generic point-level deep network for segmentation and classification. It adds a hierarchical set abstraction on the basis of the original PointNet network to extract better local features. PointNet +  + (shown in Fig. 2a) consists of two parts—an encoder of multiple feature abstractions and a decoder that can serve both segmentation and classification purposes. The feature abstraction module includes the Sampling, the Grouping, and the original PointNet Layer. The decoder can be designed to satisfy either the need of semantic segmentation or the overall point cloud classification. In the decoder for semantic segmentation, the sparse high-level point-level features are gradually propagated to the original point space by interpolation to achieve point-level segmentation.

DGCNN [60] (Dynamic Graph Convolutional Neural Network) uses Multi-layer Perceptrons (MLPs) to construct a dynamic graph convolution network, and extracts the deep local information association in the feature space by means of graph filtering. Figure 2b summarizes the main architecture of DGCNN; the backbone of DGCNN is a simple but effective EdgeConv block, which takes k-nearest neighbors in the feature space to construct a local neighborhood graph, and aggregates features through convolution operations and pooling to update point features. By cascading the EdgeConv blocks, the connectivity and shape of the feature graph can be learned by the network itself, which improves the performance of point cloud semantic segmentation. The state-of-the-art performance has made DGCNN a benchmark network for point cloud semantic segmentation.

ASIS [63] (Associatively Segmenting Instances and Semantics) is a pioneering work in the field of general-purpose dual-function point cloud segmentation network. As shown in Fig. 2c, it has an end-to-end dual-function deep network for point cloud data. ASIS can simultaneously perform semantic segmentation and instance segmentation by using two pathways with interconnections. The semantic segmentation pathway distinguishes different semantic labels of points in a point cloud, while the instance segmentation pathway clearly distinguishes different instances in each semantic class.Specifically, ASIS first extract features separately in the two task pathways, and after interconnections on the two pathways, the two segmentation tasks are together constrained by the loss functions.

PlantNet [61] is a dual-function segmentation network specialized for multi-species crop point clouds; it can simultaneously conduct organ semantic segmentation and leaf instance segmentation. PlantNet also adopts a dual-pathway architecture (the main architecture is shown in Fig. 2d), which integrates a shared encoder, a biologically inspired double-stream decoder, several Local Feature Extraction Operations (LFEOs) based on EdgeConvs, a Feature Fusion Module (FFM), and a network backend based on the spatial attention mechanism. On a crop point cloud dataset of three species, PlantNet claimed better results than several other networks.

PSegNet [67] is also a dual-function deep learning network designed for segmenting point cloud data of multiple crop species. It achieved satisfactory organ semantic segmentation results and leaf instance segmentation results for tomato, tobacco, and sorghum plants. The network (shown in Fig. 2e) begins with a shared encoder, the key of which is a component called Local Feature Extraction Module (LFEM) for local feature extraction. A Dual-Granularity Feature Fusion Module (DGFFM) is designed to blend two feature streams in the middle part. The third part features a typical dual-pathway structure, in which the calculation incorporates both spatial attention and channel attention. The two different pathways ultimately achieves semantic and instance segmentations under different loss functions, respectively.

## Experiments

This section explains the details of the comprehensive experiments. Sub-section "Dataset" shows how we form the plant point cloud dataset for training and testing, and explains the data augmentation procedures for different down-sampling strategies. Sub-section "Network training and testing" shows the details of the network training and testing. Sub-section "Quantitative evaluation metrics" defined the quantitative evaluation metrics in the experiments.

### Dataset

The crop point cloud dataset used in this study originates from [72, 73]. The dataset is obtained by imaging plant samples with a non-contact 3D scanner, and has a high scanning accuracy (error less than 1 mm). The dataset contains point clouds of tomato, tobacco and sorghum in 3 to 5 growth stages for about 20 days, including a total of 312 tomato point clouds, 105 tobacco point clouds and 129 sorghum point clouds. Tomato and tobacco are dicotyledonous plants, sorghum is monocotyledonous plant, and the three kinds of crops have different shapes. The diversity and difference in 3D plant shape pose big challenges for conducting organ segmentation task on this dataset.

Since the original data set does not have point-level labels, we continue to use the labeling tool from [74] to label the dataset with semantic and instance labels. Our manually labeled dataset is also used in [61] and [67]. Each species in the dataset has two semantic classes: the leaves and the stem system, and the leaves class has an separate instance label for each single leaf, respectively. Thus, for the final dataset, a total of 6 organ semantic classes were set for 3 species of crops, which means $C = 6$. There are no instances of the stems on the crops, because all stem segments of each plant are fully connected. The leaf instance labels of the three varieties are set on the basis of leaf semantic labels. We divide the dataset into a training set and a testing set according to a ratio of 2:1. In order to strengthen the training of the segmentation network, we designed different data augmentation strategies for five down-sampling methods, respectively. The training data and testing data are augmented by 10 times with randomness. The down-sampled dataset after each sampling strategy has 5460 point clouds (i.e., the total dataset includes 5460*5 = 27300 point clouds), and each point cloud is fixed at 4096 points. Taking FPS as an example, the first point of each FPS iteration is randomly chosen and FPS is independently applied on each original point cloud for 10 times, introducing high diversity for the training process.

### Network training and testing

All experiments in this research were carried out on a server running the Ubuntu 20.04 operating system, with a 24-core AMD 3960X CPU, 128 GB DDR4 memory, and three paralleled NVIDIA RTX 2080Ti GPUs. In order to achieve the optimal training effect for each point cloud segmentation network, we deploy all networks by the TensorFlow 1.13.1/1.9.1 environment.

In order to assure fair comparisons, we tried our best to use the same set of hyperparameters in all network training. During the training phase, the batch size was set to 10, the initial learning rate was 0.002, and the learning rate dropped by 30% every 10 Epochs. The networks were all optimized using the Adam solver, and the Momentum was fixed to 0.9. All networks uniformly trained for 200 epochs, and the model weights with the lowest validation loss in the last 100 epochs was selected as the adopted model. The batch size was fixed at 1 for all network testing processes. For the three dual-function segmentation networks—ASIS [63], PlantNet [61] and PSegNet [67], they also needs the Meanshift process (bandwidth = 0.6) to cluster the feature space for instance loss calculation. If the number of points in an instance feature cluster is less than 1% of the average number of points in an instance, the instance cluster is discarded to avoid over-segmentation. Other hyperparameters and configurations in these networks that are not explicitly introduced are the same as those in the respective original papers or source codes.

### Quantitative evaluation metrics

In the experiments of this paper, we use PointNet +  + and DGCNN to perform semantic segmentation tasks on three types of crops, and use ASIS, PlantNet, and PSegNet networks for both semantic and instance segmentation tasks. For the semantic segmentation task, we compute four fundamental quantitative metrics: *Precision*、*Recall*、*F1* and Intersection over Union (*IoU*). For these four semantic metrics, higher scores mean better segmentation performance. *Precision* is used to measure the proportion of true points in the predicted points (True Positive, TP) in a certain category to the total predicted points of the same category (True Positive + False Positive). *Recall* measures the proportion of the true points in the predicted points (TP) in a certain category to the total true points in that category. all points belonging to a certain category that the network can correctly predict (True Positive + False Negative). *Precision* and *Recall* are sometimes contradictory. Neither of them can make an overall and complete evaluation of the semantic segmentation performance alone. They must be combined with other evaluation measures to form a comprehensive evaluation. *F1* is the harmonic mean of *Precision* and *Recall* with a value ranging from 0 to 1, so it is a commonly used comprehensive evaluation. For each semantic category (class), *IoU* is a standard comprehensive performance measure for segmentation. It is used to measure the degree of overlap between the network prediction results and the Ground Truth (GT); its value also ranges from 0 to 1, a higher value indicates a better alignment between the predicted results and GT. The equation definitions of these four semantic quantitative metrics are as follows:

4 $${\text{Precision}} = \frac{TP}{{TP + FP}},$$

5 $${\text{Recall}} = \frac{TP}{{TP + FN}},$$

6 $$F1 = 2 \cdot \frac{{{\text{Precision}} \cdot {\text{Recall}}}}{{{\text{Precison}} + {\text{Recall}}}},$$

7 $$IoU = \frac{TP}{{TP + FP + FN}}.$$

For the instance segmentation task, we first choose mean coverage (*mCov*), mean weighted coverage (*mWCov*) [75–77] as comprehensive evaluation criteria at the point level. The value range of *mCov* is between 0 and 1, where a higher value indicates better performance. *mWCov* is a weighted version of *mCov*. On the basis of *mCov*, *mWCov* performs weighted calculation according to its percentage of the instance points in the total class. The equations of the two coverage metrics are as follows:

8 $$mCov(I,P) = \frac{1}{\left| I \right|}\sum\limits_{m = 1}^{\left| I \right|} {\mathop {\max }\limits_{n} } IoU(I_{m} ,P_{n} ),$$

9 $$mWCov(I,P) = \sum\limits_{m = 1}^{\left| I \right|} {\omega_{m} \mathop {\max }\limits_{n} IoU(I_{m} ,P_{n} )} ,$$

10 $$\omega_{m} = I_{m} /\sum\limits_{k = 1}^{\left| I \right|} {I_{k} ,}$$

where $I_{m}$ denotes the number of points contained in the region of the *m-th* Ground Truth instance. $P_{n}$ represents the *n*-th predicted instance region, and $\left| I \right|$ is the number of all instances contained in a true semantic category. In addition to the above two point-level evaluation criteria, *mPrec* and *mRec* are also used to evaluate the completeness of the network to predict the instance. They are defined as follows:

11 $${\text{mPrec}} = \frac{1}{{C_{ins} }}\sum\limits_{i = 1}^{{C_{ins} }} {\frac{{\left| {TP_{i}^{ins} } \right|}}{{\left| {P_{i}^{ins} } \right|}},}$$

12 $${\text{mRec}} = \frac{1}{{C_{ins} }}\sum\limits_{i = 1}^{{C_{ins} }} {\frac{{\left| {TP_{i}^{ins} } \right|}}{{\left| {G_{i}^{ins} } \right|}},}$$

where $\left| {TP_{i}^{ins} } \right|$ is the number of instances predicted by the network that belong to the *i-th* semantic class and has an *IoU* greater than 0.5; $\left| {P_{i}^{ins} } \right|$ is the total number of instances contained in the *i-th* semantic class predicted by the network. $\left| {G_{i}^{ins} } \right|$ is the number of instances contained in the *i-th* semantic category in GT. $C_{ins}$ means the number of semantic classes that have instances. In the dataset adopted in this paper, only the leaves of three species have instance labels, so $C_{ins}$ in Eqs. (11, 12) is 3.

In order to better quantitatively evaluate the effects of different down-sampling strategies on different mainstream point cloud deep learning networks, respectively, it is still difficult to make a comprehensive judgment based solely on the quantitative evaluation metrics mentioned above. This is because the mixture of multiple crop types, down-sampling strategies, networks, and quantitative metrics will result in a very complicated data table, which hinders us from making a clear comparison. Therefore, we designed a scoring method to comprehensively leverage the quantitative measures obtained by each down-sampling strategy under different deep learning networks. Our scoring rules are as follows:

(i) We ran cross-tests for five down-sampling strategies on the five different networks. The scores are calculated according to the values of the quantitative measures calculated by Eqs. (4–12).
(ii) For semantic segmentation under the same network, we first rank the 5 down-sampling strategies in each single quantitative metric. Then, corresponding scores are assigned based on the rankings. The assignment of scores only focuses on the top three quantitative measures. For *Precision* and *Recall*, since neither of them provide a comprehensive evaluation of network semantic segmentation, their scores are set lower than *F1* and *IoU*. For each comparison, *Precision* and *Recall* are scored 3, 2, and 1 points for the top three in the five strategies, while the *F1-score* and *IoU* are scored 6, 4, and 2 for the top three, respectively.
(iii) For instance segmentation under the same network, the approach is similar to semantic segmentation. For ${\text{mPrec}}$ and ${\text{mRec}}$, since neither of them provide a comprehensive evaluation of network semantic segmentation, their scores are set lower than $mCov$ and ${\text{mWCov}}$. For each comparison, ${\text{mPrec}}$ and ${\text{mRec}}$ are scored 3, 2, and 1 points for the top three in the five strategies, while $mCov$ and ${\text{mWCov}}$ are scored 6, 4, and 2 for the top three, respectively.
(iv) After steps (ii) and (iii) are completed, under each network, we add up the separate scores of each down-sampling strategy to get the total score. There are two situations for the five types of networks. First, PointNet +  + and DGCNN are single-function semantic segmentation networks. Therefore, we only compare the scores of the two networks added up from four semantic quantitative metrics, and the total score for each down-sampling strategy is only discussed in the semantic segmentation context. Second, for dual-function networks including ASIS, PlantNet, and PSegNet, the total score of each down-sampling strategy is added up from the four semantic segmentation metric scores and four instance segmentation metric scores. And the total score of the second situation reveals both semantic and instance segmentation performances.
(v) Except from the above steps, we also calculate the difference of each quantitative metric value of a strategy with the best value of that metric, and then calculate the average difference across all quantitative metrics. This average difference is denoted by *AveDiff*. For example, given the RS strategy on PointNet +  + network obtains *Precision, Recall*, *F1*, *IoU* at 85%, 84%, 86%, and 84%, respectively; and the best performance values across all strategies are 86%, 84%, 87%, and 85%, respectively; the *AveDiff* for RS on PointNet +  + is (1% + 0% + 1% + 1%)/4 = 0.75%. The *Score* value can intuitively represent the ranking of segmentation performance, and the *AveDiff* value can show the real difference in performance between each down-sampling strategy and the best individual, which helps to reflect the performance stability.

## Results

This section gives the experimental results. The comparative quantitative results are given in sub-section "Quantitative results". The qualitative results are visualized in sub-section "Qualitative results". The summary and suggestions regarding to the experiments are given in the last sub-section.

### Quantitative results

The quantitative experiments in this sub-section first evaluate the performances of down-sampling strategies on two point cloud semantic segmentation networks—PointNet +  + and DGCNN. Then, the comparative experiments on the other three dual-function segmentation networks—ASIS, PlantNet, and PSegNet are carried out. Finally, we summarize the experimental results and observations for both types of the networks.

Table 2 shows the quantitative semantic segmentation results on the PointNet +  + network under five down-sampling strategies. The results not only include the four fundamental semantic segmentation metrics, but also include the final scores and AveDiffs. The best result of each measure is highlighted in bold, and the second-best is underlined. Each semantic metric value in Table 2 is obtained by averaging of four independent experiments on the complete dataset. From Table 2, it is evident that UVS achieves the best result across all strategies in the semantic segmentation task of PointNet +  + , with a significant advantage. The overall performance of 3DEPS ranks second, with an AveDiff of 0.5%. The strategy with the lowest score is FPS, which has a 1.10% average performance difference with UVS.

**Table 2:** Quantitative comparison of the semantic segmentation performance across five down-sampling strategies on PointNet +  +

PointNet + +	Semantic Segmentation (%)				Score	AveDiff (%)
	Precision	Recall	F1	IoU		
FPS	85.38	84.62	84.96	75.77	0	1.10
RS	85.29	84.81	85.01	76.00	2	1.01
UVS	**86.07**	**86.15**	**85.90**	**77.03**	**18**	**0.00**
VFPS	85.55	84.95	85.08	75.91	4	0.71
3DEPS	85.64	86.05	85.74	76.80	12	0.50

Table 3 shows the quantitative comparison of the semantic segmentation results on the DGCNN network under five different down-sampling strategies. In Table 3, the RS strategy achieves the best on most of the metrics in the semantic segmentation task of DGCNN. The 3DEPS ranks second on the overall performance, with only a little difference on performance from RS. On the AveDiff index, RS and 3DEPS have significant advantages in segmentation stability compared to the other three strategies.

**Table 3:** Quantitative comparison of the semantic segmentation performance across five down-sampling strategies on DGVNN

DGCNN	Semantic Segmentation (%)				Score	AveDiff (%)
	Precision	Recall	F1	IoU		
FPS	92.03	90.50	91.22	84.85	0	1.71
RS	**93.23**	92.09	**92.64**	**87.04**	**16**	**0.11**
UVS	92.53	91.75	92.13	86.25	5	0.70
VFPS	91.98	92.10	92.04	86.11	2	0.81
3DEPS	92.67	**92.56**	92.60	86.93	13	0.17

From the experimental results of the two single-function networks, the down-sampling strategy with the highest score on PointNet +  + is UVS, and the strategy with the highest score on DGCNN is RS. The FPS performs worst on both networks. The second-best performers in score of the two networks are both 3DEPS, and according to the AveDiff value, 3DEPS has only a small gap with the best performer in both cases. The UVS method requires parameter tuning on the size of voxelization, which may not suitable for automated processing. Although the RS method performs well in DGCNN, it is not quite suitable for PointNet +  + . In summary, 3DEPS can achieve high-quality and stable results on the single-function semantic segmentation networks, and the commonly used FPS down-sampling tends to have the worst result across the five strategies.

Table 4 shows the quantitative semantic and instance segmentation results of five down-sampling strategies on the ASIS network. The 10 quantitative metrics listed in Table 4 include four semantic segmentation measures and four instance segmentation measures listed in "Experiments" section and also include the total score and AveDiff on both segmentation tasks. The best result for each metric in the table is in bold and the second best is underlined. Each semantic segmentation metric in Table 4 is the average value of six different semantic classes of stems or leaves in the dataset obtained from four independent experiments, and each instance segmentation metric is the average of leaf instances of all species in the dataset obtained from four independent experiments (the stem system is treated as a whole without instance concept). The score and AveDiff cover the results of all semantic and instance metrics.

**Table 4:** Quantitative comparison of both semantic segmentation and instance segmentation performances across five down-sampling strategies on the ASIS network

ASIS	Semantic Segmentation (%)				Instance Segmentation (%)				Score	AveDiff (%)
	**Precision**	**Recall**	**F1**	**IoU**	**Cov**	**WCov**	**mPrec**	**mRec**		
FPS	79.77	81.44	79.62	67.24	63.46	70.37	69.58	**58.41**	14	1.96
RS	82.67	82.09	82.04	70.28	62.85	70.34	67.73	53.56	13	1.88
UVS	83.97	81.09	81.93	70.18	61.29	69.10	68.73	52.15	3	2.14
VFPS	**84.33**	81.03	82.08	**70.66**	62.20	69.88	67.90	51.73	13	1.92
3DEPS	83.33	**82.48**	**82.34**	70.24	**64.01**	**71.92**	**71.45**	54.62	**29**	**0.64**

Based on Table 4, 3DEPS has the best performance on ASIS. Although FPS has achieved the second place in the total score, it has a big gap with 3DEPS according to the AveDiff. On ASIS network, UVS has the lowest score. All other down-sampling strategies have significant performance gaps compared to 3DEPS, with all their AveDiff values above 1.80%. The experiment shows that 3DEPS may be the most suitable down-sampling strategy for the ASIS dual-function segmentation network.

Table 5 shows the quantitative semantic and instance segmentation results of five down-sampling strategies on the PlantNet network. In Table 5, 3DEPS and UVS obtain the highest score. On the semantic segmentation task, 3DEPS achieves the best results, and UVS ranks second. UVS performs the best on the task of instance segmentation, followed by RS and 3DEPS. In total, both UVS and 3DEPS can achieve satisfactory results on the PlantNet network. Although RS ranks third, the gap with the best is not obvious (the AveDiff of RS is only 0.33%).

**Table 5:** Quantitative comparison of both semantic segmentation and instance segmentation performances across five down-sampling strategies on the PlantNet network

PlantNet	Semantic Segmentation (%)				Instance Segmentation (%)				Score	AveDiff (%)
	**Precision**	**Recall**	**F1**	**IoU**	**Cov**	**WCov**	**mPrec**	**mRec**		
FPS	91.83	89.56	90.63	83.22	77.27	83.23	81.89	**76.19**	3	2.54
RS	93.38	91.87	92.60	86.66	79.38	86.25	**85.43**	75.97	12	0.33
UVS	93.66	92.35	92.99	87.21	**79.72**	**86.44**	84.83	75.17	**25**	0.22
VFPS	**93.67**	91.84	92.77	86.88	77.90	85.55	81.61	73.70	7	1.28
3DEPS	93.61	**92.45**	**93.02**	**87.29**	79.22	86.25	85.10	75.60	**25**	**0.20**

Table 6 shows the quantitative semantic and instance segmentation results of five down-sampling strategies on the PSegNet. In Table 6, UVS achieves overwhelming advantages on both semantic and instance segmentation tasks. VFPS and 3DEPS are tied for second place for the score metric, but both have a significant gap with UVS. The FPS has the lowest score across all strategies. The voxel down-sampling strategies (UVS and VFPS) seem to be more suitable for the PSegNet network.

**Table 6:** Quantitative comparison of both semantic segmentation and instance segmentation performances across five down-sampling strategies on the PSegNet network

PSegNet	Semantic Segmentation (%)				Instance Segmentation (%)				Score	AveDiff (%)
	Precision	Recall	F1	IoU	Cov	WCov	mPrec	mRec		
FPS	90.03	89.38	89.68	81.90	79.63	85.50	84.30	**78.18**	7	2.12
RS	91.71	91.04	91.35	84.62	78.79	85.67	83.54	72.80	0	2.01
UVS	**93.10**	**92.62**	**92.67**	**86.78**	**80.12**	**86.77**	**85.37**	74.99	**35**	**0.39**
VFPS	92.26	92.14	92.18	85.99	78.21	85.67	85.05	72.27	15	1.48
3DEPS	92.24	91.86	92.04	85.74	79.03	85.92	84.37	73.48	15	1.36

Based on the quantitative experimental results of the three dual-function networks, 3DEPS achieves satisfactory scores on both ASIS and PlantNet. UVS achieves high scores on PlantNet and PSegNet. In general, 3DEPS performs best on dual-function networks across all strategies compared; but its advantage over the second best is not significant, given UVS’s big advantage on the state-of-the-art PSegNet. Therefore, for the state-of-the-art dual-function segmentation networks, carefully selecting an appropriate down-sampling strategy can further improve the segmentation performance. However, the process of manual parameter tuning and selection of the down-sampling strategy can be quite time-consuming. If one does not pursue the optimal ceiling of the segmentation performance on a particular network, 3DEPS may be a good choice due to its sub-optimal and stable performance.

### Qualitative results

**Fig. 3:**
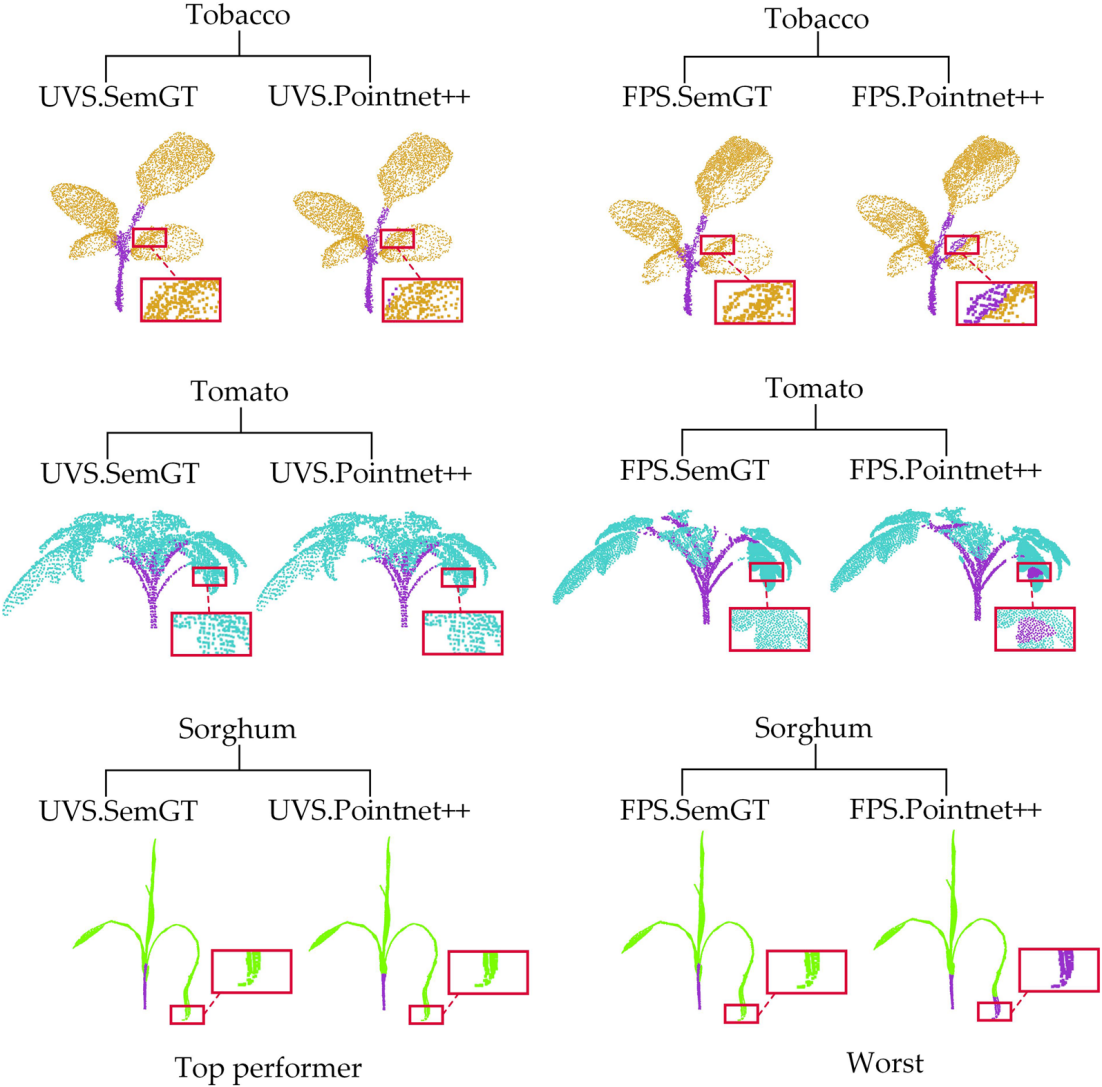
Comparison of the best and worst down-sampling strategies for qualitative segmentation results on the PointNet +  + network. The first row shows the segmentation results of 3 types of crop samples using UVS, and the second row shows the plant segmentation results of the same samples using FPS. Each crop point cloud segmented by PointNet +  + is contrasted with its corresponding Ground Truth, and the red boxes are zoomed in areas for better visualization

Figure 3 compares the qualitative semantic segmentation results from different strategies on three different plant species upon the PointNet +  + network. According to Table 2, the overall best down-sampling strategy for the PointNet +  + network is UVS, while the worst is FPS. In order to clearly demonstrate the difference in semantic segmentation performance between down-sampling strategies, we specifically compare the segmentation results of the best down-sampling strategy (UVS) and the worst down-sampling strategy (FPS). The qualitative comparison in Fig. 3 shows that there is an evident difference between the results obtained with UVS and FPS. The test samples after FPS down-sampling tends to produce segmentation errors at the intersection of leaves and stems and at the tips of long leaves.

**Fig. 4:**
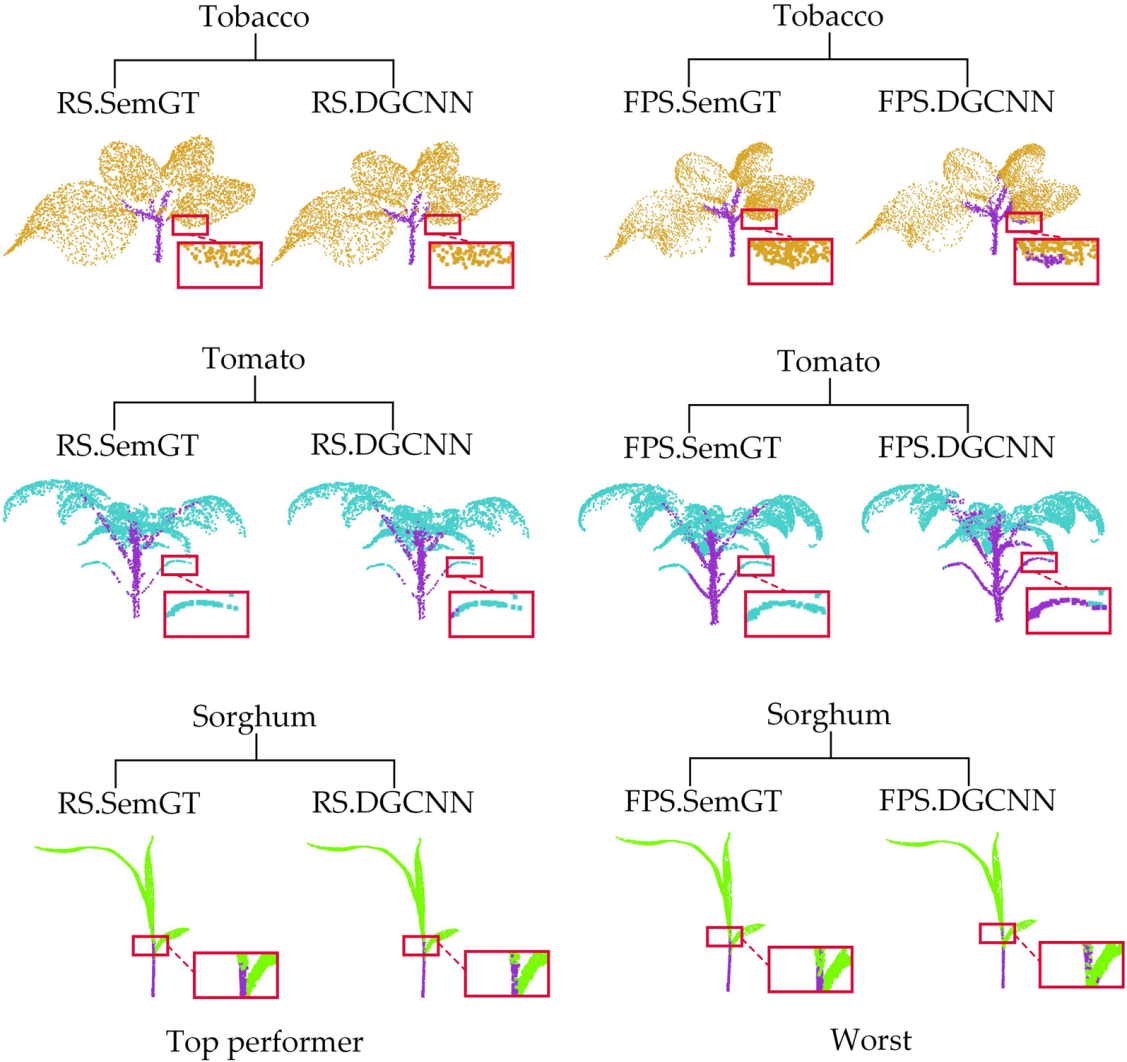
Comparison of the best and worst down-sampling strategies for qualitative segmentation results on the DGCNN network. The first row shows the segmentation results of 3 types of crop samples using RS, and the second row shows the plant segmentation results of the same samples using FPS. Each crop point cloud segmented by DGCNN is contrasted with its corresponding Ground Truth, and the red boxes are zoomed in areas for better visualization

Figure 4 compares qualitative results of semantic segmentation from different strategies on three different plant species upon the DGCNN network. According to Table 3, the overall best down-sampling strategy for DGCNN is RS, and the worst is FPS. We then compare the best result (RS) and the worst result (FPS) for each test sample in Fig. 4, it can be clearly seen that the test samples after RS down-sampling show much better qualitative segmentation results than FPS with few segmentation errors. FPS tends to generate errors under DGCNN at the leaf edges and the connection between leaf and stem.

**Fig. 5:**
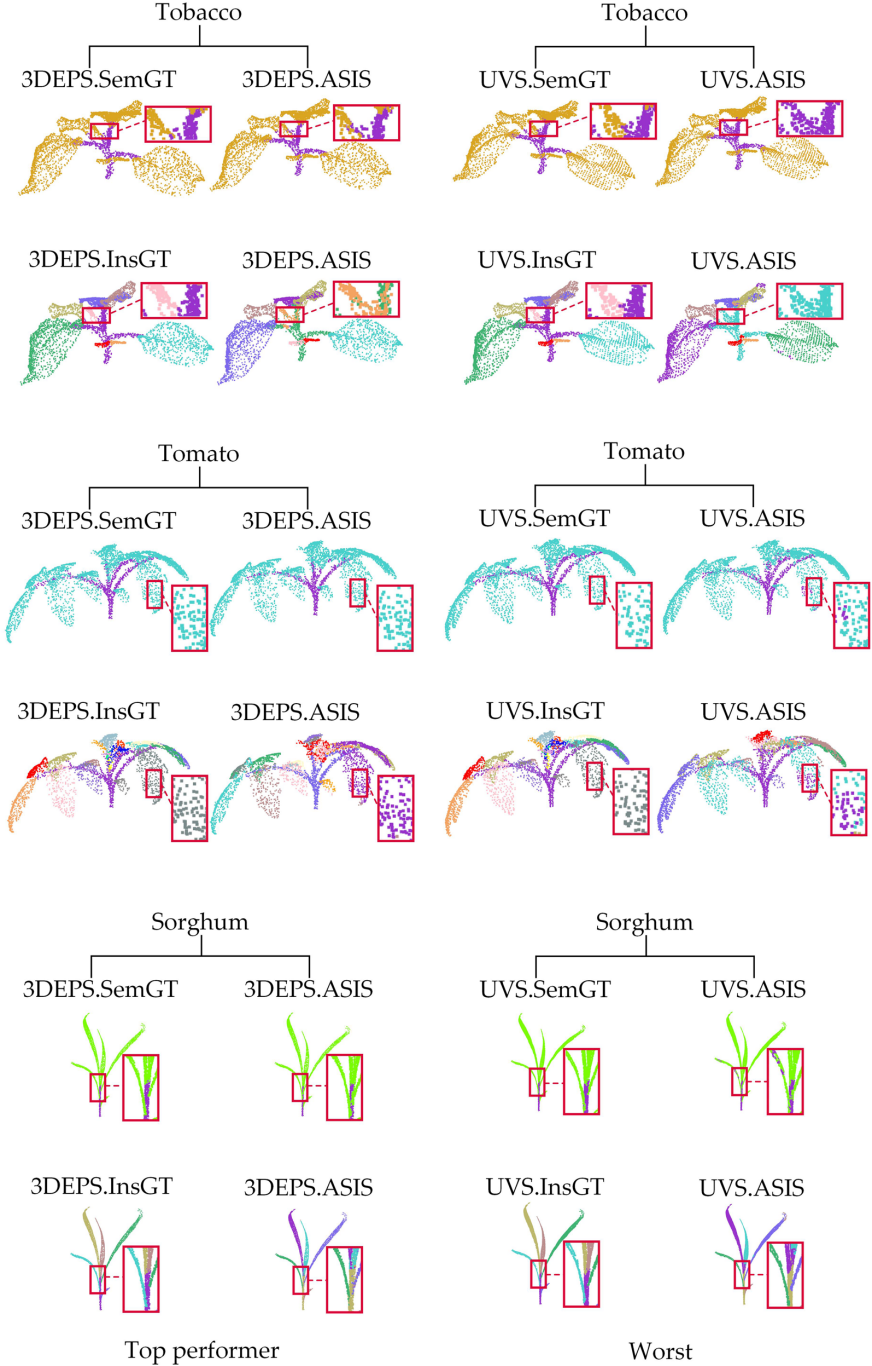
Comparison of the best and worst down-sampling strategies for qualitative segmentation results on the ASIS network. The first and second rows show the semantic segmentation and instance segmentation results, respectively, of three different samples using the 3DEPS strategy on ASIS. The third and fourth rows show the semantic segmentation and instance segmentation results, respectively, of the same three plant samples using the UVS strategy on ASIS. Each segmented plant point cloud is compared with its corresponding Ground Truth, and the red boxes are zoomed in areas for better visualization. Please note that for the instance segmentation, different colors are only used to distinguish between different adjacent leaf organ instances, and there is no correspondence between leaf color and leaf index

Figure 5 shows the qualitative results of semantic segmentation and instance segmentation of 3 different types of plants on the ASIS network. From Table 4 it can be seen that the overall best down-sampling strategy under ASIS is 3DEPS, and the worst is UVS. To clearly visualize the gap in segmentation performance across different strategies, we only compare the best down-sampling segmentation result (3DEPS) and the worst down-sampling segmentation result (UVS) for the same test samples on both segmentation tasks. From Fig. 5, it can be observed that there is a evident difference between the 3DEPS results and the UVS results. The plant samples after 3DEPS down-sampling show satisfactory semantic and instance segmentation results by ASIS, and the plant samples after UVS down-sampling have multiple semantic and instance segmentation errors at some leaf tips, small leaves, and leaf-stem connections.

**Fig. 6:**
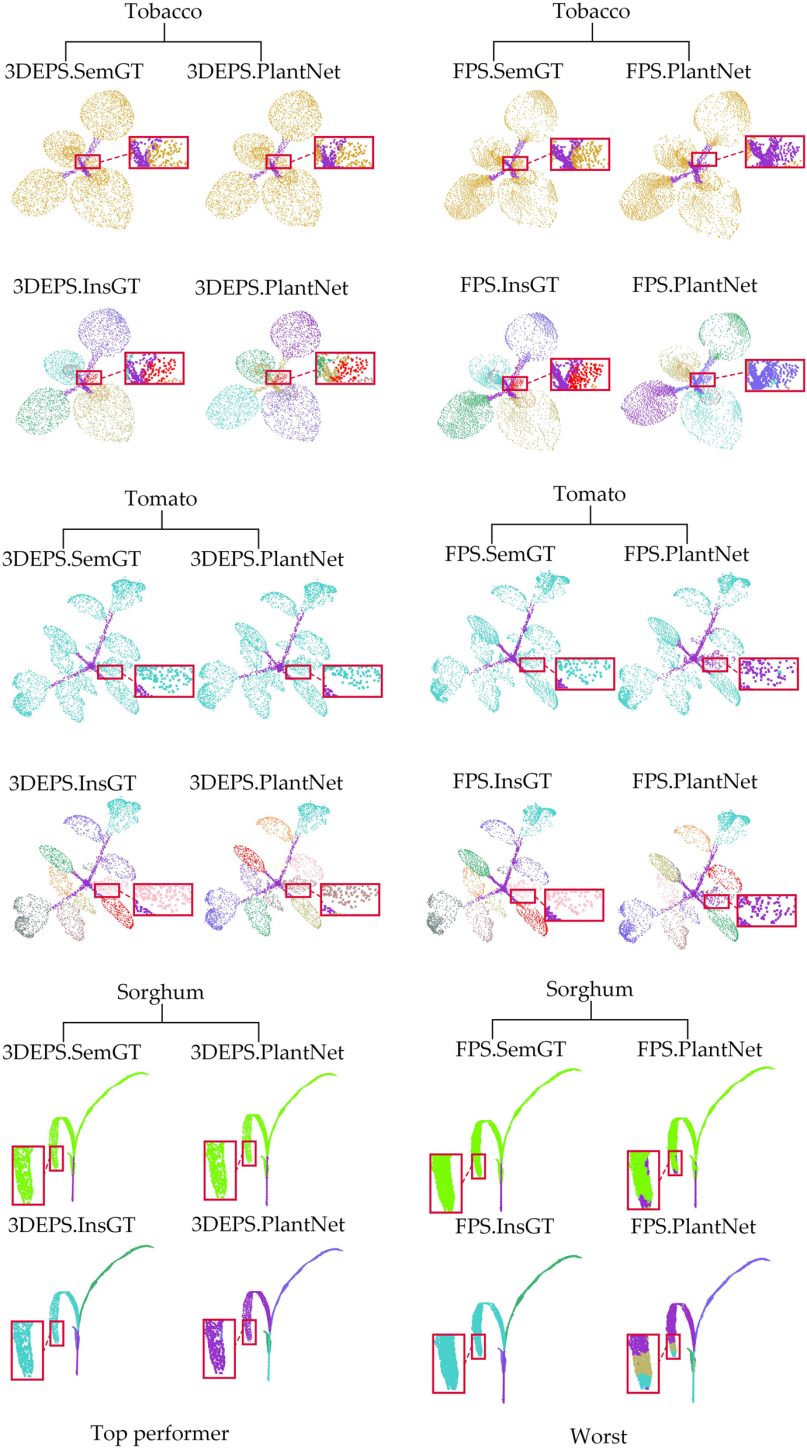
Comparison of the best and worst down-sampling strategies for qualitative segmentation results on the PlantNet network. The first and second rows show the semantic segmentation results and instance segmentation results, respectively, of three different samples using the 3DEPS strategy on PlantNet. The third and fourth rows show the semantic segmentation results and instance segmentation results, respectively, for the same three plant samples using the FPS down-sampling strategy on PlantNet. Each segmented crop point cloud is contrasted with its corresponding Ground Truth, and the red boxes are zoomed in areas for better visualization. Please note that for the instance segmentation, different colors are only used to distinguish between different adjacent leaf organ instances, and there is no correspondence between leaf color and leaf index

Figure 6 shows the qualitative results of semantic segmentation and instance segmentation of 3 different types of plants on the PlantNet network. According to Table 5, the overall best down-sampling strategy on this network is 3DEPS, while the worst is FPS. In order to clearly demonstrate the gap across different down-sampling strategies, we compare the results of the same test sample from the best down-sampling segmentation (3DEPS) and the worst down-sampling segmentation (FPS) for both semantic segmentation and instance segmentation tasks, respectively. In Fig. 6, the 3DEPS shows satisfactory results on PlantNet on both segmentation tasks, while FPS tends to have segmentation errors at leaf tip (especially the sorghum leaf) and at small leaves.

**Fig. 7:**
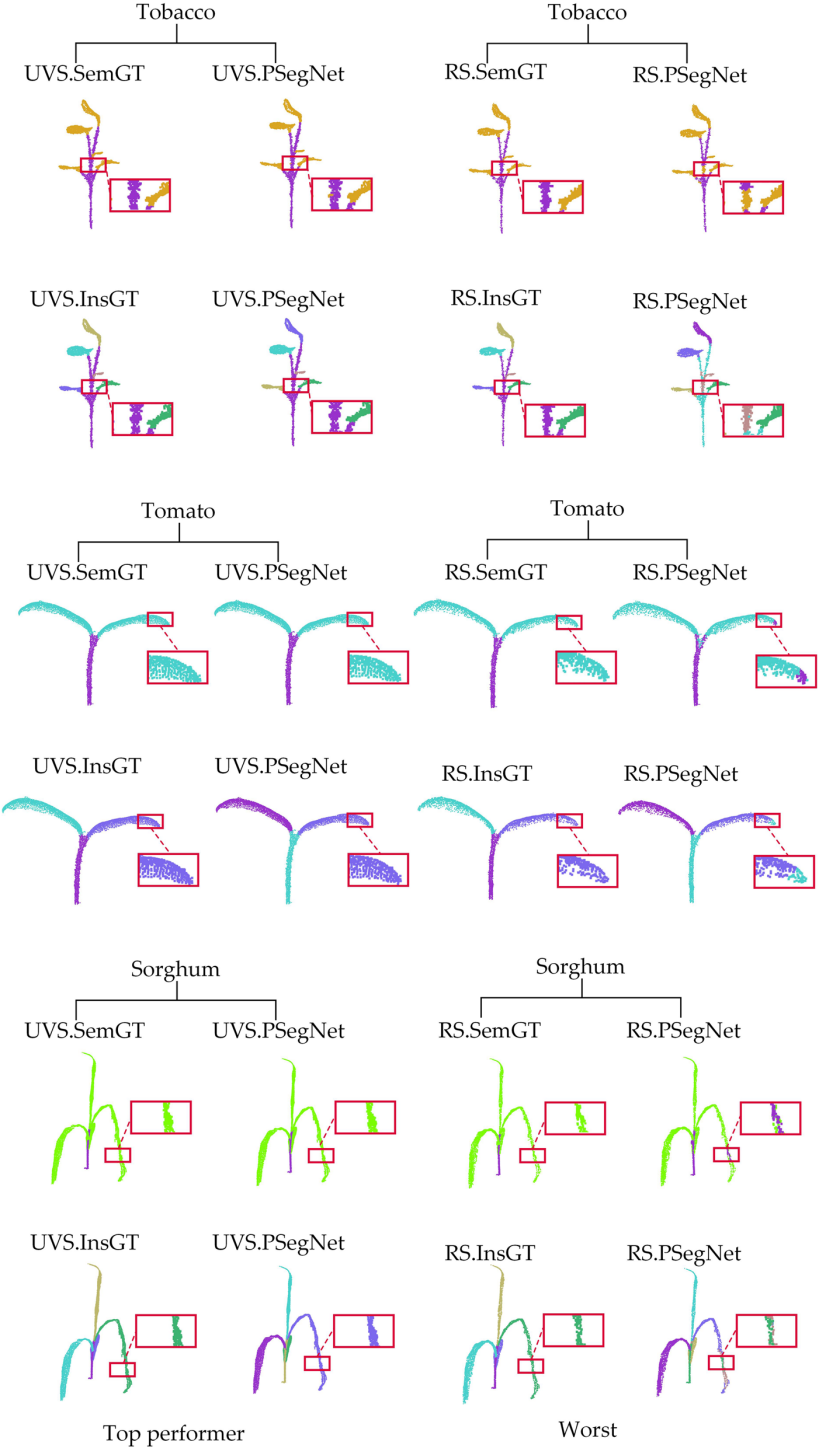
Comparison of the best and worst down-sampling strategies for qualitative segmentation results on the PSegNet network. The first row and the second row are the semantic segmentation results and instance segmentation results of the three samples using the UVS down-sampling strategy on PSegNet, respectively. The third and fourth rows show the results of semantic segmentation and instance segmentation, respectively, for the same three samples using the RS strategy on PSegNet. Each segmented crop point cloud is contrasted with its corresponding GT, and the red boxes are zoomed in areas for better visualization. Please note that for the instance segmentation, different colors are only used to distinguish between different adjacent leaf organ instances, and there is no correspondence between leaf color and leaf index

Figure 7 shows the qualitative results of semantic segmentation and instance segmentation of 3 different types of plants on the PSegNet network. According to Table 6, the overall best down-sampling strategy in this network is UVS, while the worst is RS. We compare the results of the same test sample from the best down-sampling segmentation (UVS) and the worst down-sampling segmentation (RS) for both semantic segmentation and instance segmentation tasks, respectively. From Fig. 7, it can be observed that the plant samples after UVS exhibit satisfactory results in terms of both semantic and instance segmentation on PSegNet. The plant samples after RS down-sampling tend to exhibit segmentation errors, particularly at the end tip of tomato leaves and in the middle of long organs.

By analyzing the qualitative segmentation results under the down-sampling strategies, one can observe that the areas including the tip of the leaf, the stem-leaf connection, and the leaf edge are easy to produce errors. Though we can always select an appropriate down-sampling strategy to effectively avoid some segmentation errors on a specific model, it is still difficult to eliminate them all, i.e., it is still hard to pin down a down-sampling strategy that is universally perfect on all deep networks for crop point clouds.

## Summary and suggestions

**Fig. 8:**
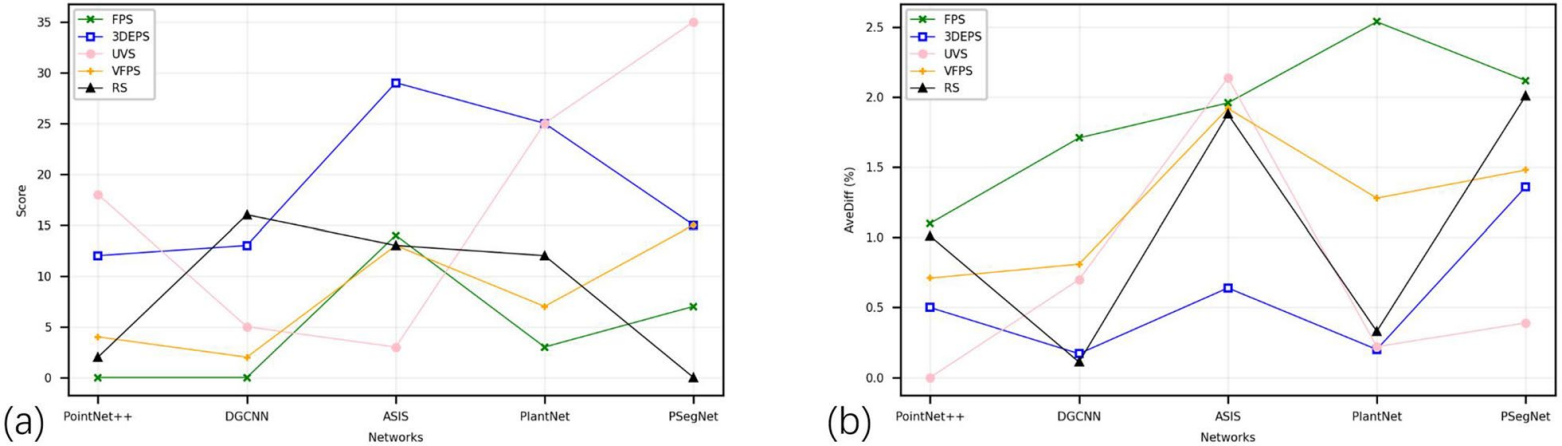
The line charts showing the Scores and AveDiff values obtained from experiments using the five down-sampling strategies on different networks. The horizontal axes of the two subgraphs both represent the types of networks. The vertical axis in **a** represents the Scores obtained (the higher the better); the vertical axis in **b** represents obtained AveDiffs, which is the lower the better

**Fig. 9:**
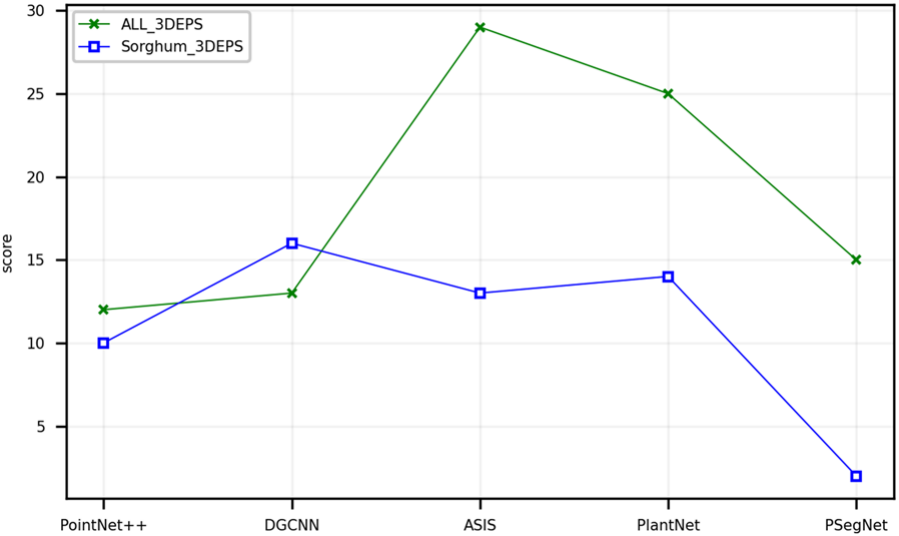
Comparison of two different plant datasets sampled by 3DEPS using segmentation score. The “ALL_3DEPS” line represents the scores obtained on the original dataset, and the “Sorghum_3DEPS” line represents the scores obtained on the dataset that only contains sorghum crops. The horizontal axis in the figure indicates the network types, and the vertical axis indicates the 3DEPS scores

**Fig. 10:**
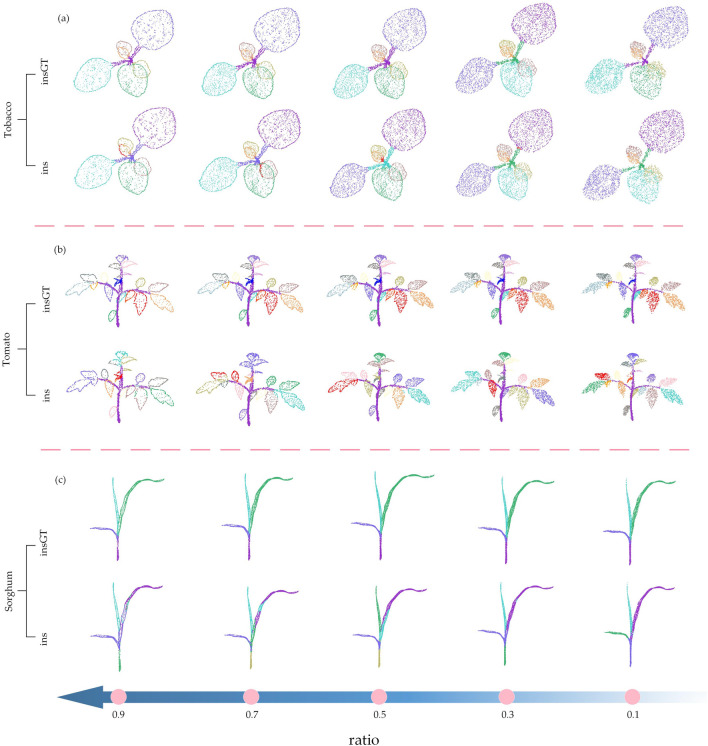
Qualitative comparison of the instance segmentation results under different 3DEPS ratio parameters on the PSegNet network. The first and the second rows are the GT and instance segmentation results of the same tobacco plant, respectively. The third and the fourth rows are the GT and segmentation results of the same tomato plant, respectively. The fifth and the sixth rows are the GT and network segmentation results of the same sorghum plant, respectively. The direction of the arrow below the figure shows the increasing direction of the ratio parameter, and segmentation results with ratios at 0.1, 0.3, 0.5, 0.7, and 0.9 are compared

**Fig. 11:**
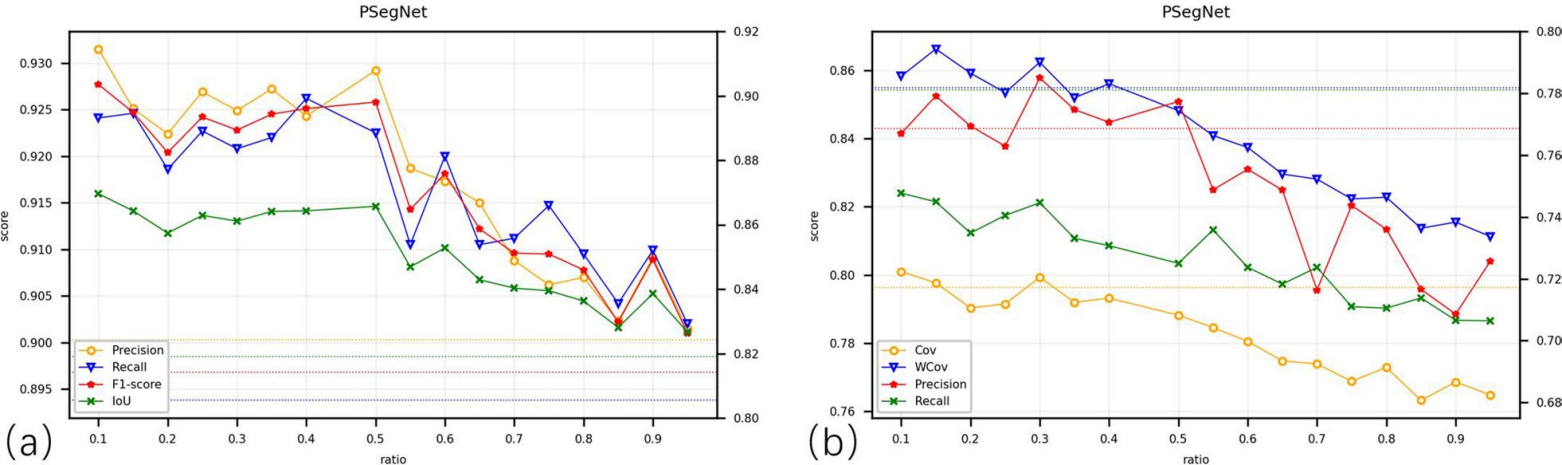
Quantitative comparison of the instance segmentation results under different 3DEPS ratio parameters on the PSegNet network. **a** represents the semantic segmentation results, and **b** represents the instance segmentation results. The horizontal dotted lines in the figure are the results of the FPS strategy (as the benchmark). It can be seen that the overall segmentation performance is affected by the ratio value. The optimal ratio parameter of the PSegNet network should not exceed 0.5

Based on the overall quantitative and qualitative experimental data, it is difficult to pin down a golden down-sampling strategy that performs best for all crop point cloud segmentation networks. Sometimes we can only find a sub-optimal down-sampling strategy based on experience and comparative experiments within a certain parameter range and application conditions. From Fig. 8a, b, it can be observed that for single-task deep networks, UVS, 3DEPS, and RS are relatively suitable down-sampling strategies. For dual-function segmentation networks, 3DEPS, UVS, and VFPS are considered better choices for sampling. 3DEPS has shown the most stable performance (and also satisfactory at most cases) in the experiments conducted in this paper. Even in cases where 3DEPS does not achieve the highest score, the margin (can be reflected from AveDiff) between 3DEPS and the top performer is not significant. Therefore, in most cases, directly applying the 3DEPS as the down-sampling strategy for networks seems to be a good choice. But 3DEPS also has two disadvantages. The first disadvantage is that for point cloud data of certain plant species and certain type of plant structure, the segmentation by 3DEPS is yet to be improved. Figure 9 shows that if the dataset only contains a monocotyledonous plant like Sorghum, the score obtained by 3DEPS can be far lower than the average 3DEPS score from the original dataset that comprises three species on the three different dual-function segmentation networks. This fact reveals that 3DEPS is more suited to dicotyledonous plants than the monocotyledonous crops that usually have long and slender leaves. The second disadvantage is that the ratio parameter of 3DEPS that controls the proportion of edge points requires extra parameter tuning experiments. Figure 10 qualitatively shows the impact of different ratio values of 3DEPS on PSegNet, and Fig. 11 quantitatively shows how the ratio of 3DEPS has clear impact on the semantic and instance segmentation performances of PSegNet. According to Fig. 11, it is currently believed that the optimal value of the ratio for a network should be between 0.1 and 0.5.

It is also interesting to notice that the five evaluated networks performed differently on the same dataset. For the single-function networks (not perform semantic segmentation), DGCNN has an evident edge over PointNet +  + on almost all quantitative metrics. And we also have compared PointNet with PointNet +  + , the most recognizable two members from the PointNet Family, on the same dataset used in this paper (the comparison is not shown in this study); PointNet +  + showed evident better segmentation performance than PointNet. If the phenotyping task is only organ semantic segmentation on a dataset containing more than two species, we suggest practioners using DGCNN instead of PointNet family. The case on dual-function network is much complicated than the single semantic segmentation. Currently, almost all dual-function networks are trained under a combined loss function that seeks a balance between the semantic segmentation task and the instance segmentation task. The direct comparison between Table 5 (PlantNet) and Table 6 (PSegNet) is hard to tell which is better, because the version of PlantNet trained under our dataset and parameter setting seems to have an edge over PSegNet on the semantics; but conversely on the instance segmentation task. A fair comparison needs fine tuning and repetitive testing on one of the network to make both networks output similar semantic segmentation results, and then compare their instance segmentation results to decide which one is better. According to the comprehensive comparative experiments in the original paper of PSegNet, PSegNet performs slightly better than PlantNet, and both of the networks are evidently superior to ASIS.

In conclusion, in order to maximize the performance of point cloud deep networks for plant phenotyping, it is important to choose a down-sampling strategy that is suitable for the network. To the best of our knowledge, there is currently no strict golden rule on down-sampling strategy for deep learning of crop point clouds.

## Discussion

Each single plant model in the dataset used in the previous section contains around 10,000–100,000 points, which are already accurate enough for applying deep learning methods. However, the performances of the evaluated down-sampling strategies and segmentation networks on very high-precision plant models are still unknown. In this section, we introduce a new high-precision point cloud dataset of Soybean plants, Soybean-MVS [78], for a further comparative evaluation. Soybean-MVS contains 102 soybean plant samples of 5 different varieties scanned using Multi-View Stereo (MVS) technique, and the data was collected during a long growth period. Because high-resolution DSLR cameras were used during image collection, the reconstructed soybean samples all have high accuracy in point density. A sample plant in Soybean-MVS can contain as much as 60,000,000 points. Due to the pure background used during data collection and the careful post-processing steps, the noise of the dataset is suppressed to a low level. With the help of the original labels provided in the dataset, we set the semantic labels of soybean plants into two classes—stem system and leaves ($C = 2$), and meanwhile we also set instance labels for each single leaf. Ten soybean samples at final growth stages lost almost all leaves; thus, we only keep the rest of 92 samples from the original dataset for network training and testing. For the 92 samples, 74 of them are used to form the training set, and the rest 18 point clouds are for testing purpose. All plant samples are augmented 10 times to increase data diversity, and each point cloud is fixed at 4096 points. Like the experimental design on the dataset in Sect. "Dataset", we ran cross-tests for five down-sampling strategies on the five different networks trained on the Soybean-MVS dataset. We also used the same quantitative metrics to evaluate the network segmentation results, and all metrics were average on three independent repeats. Therefore, the final training set of Soybean-MVS has 74*10*5 = 3700 point clouds, and the final testing set contains 18*5*3 = 270 point clouds.

**Fig. 12:**
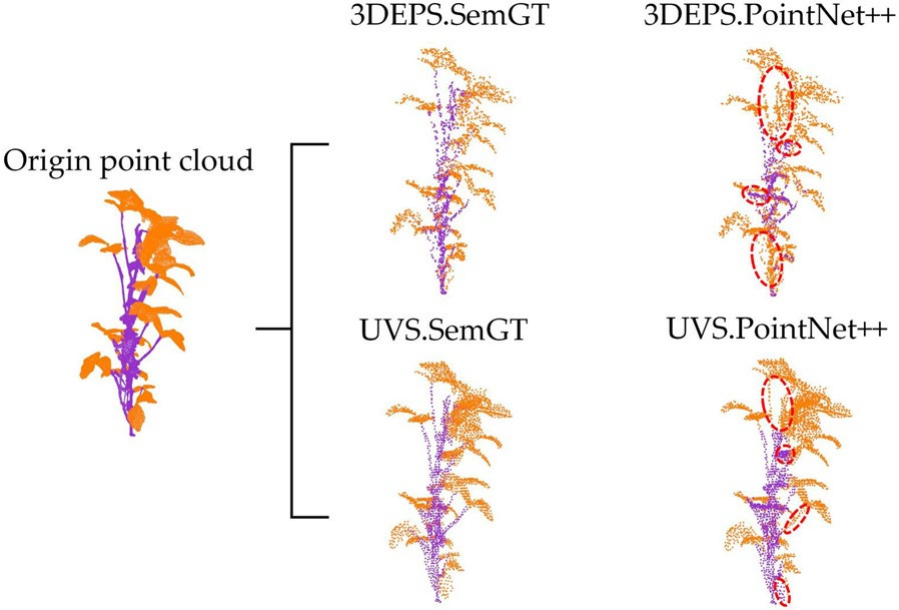
Comparison of the best and worst down-sampling strategies for qualitative segmentation results on the PointNet +  + network for Soybean-MVS dataset. Each point cloud segmented by PointNet +  + is contrasted with its corresponding Ground Truth (SemGT), and evident segmentation errors are highlighted by dotted red circles for better visualization

**Fig. 13:**
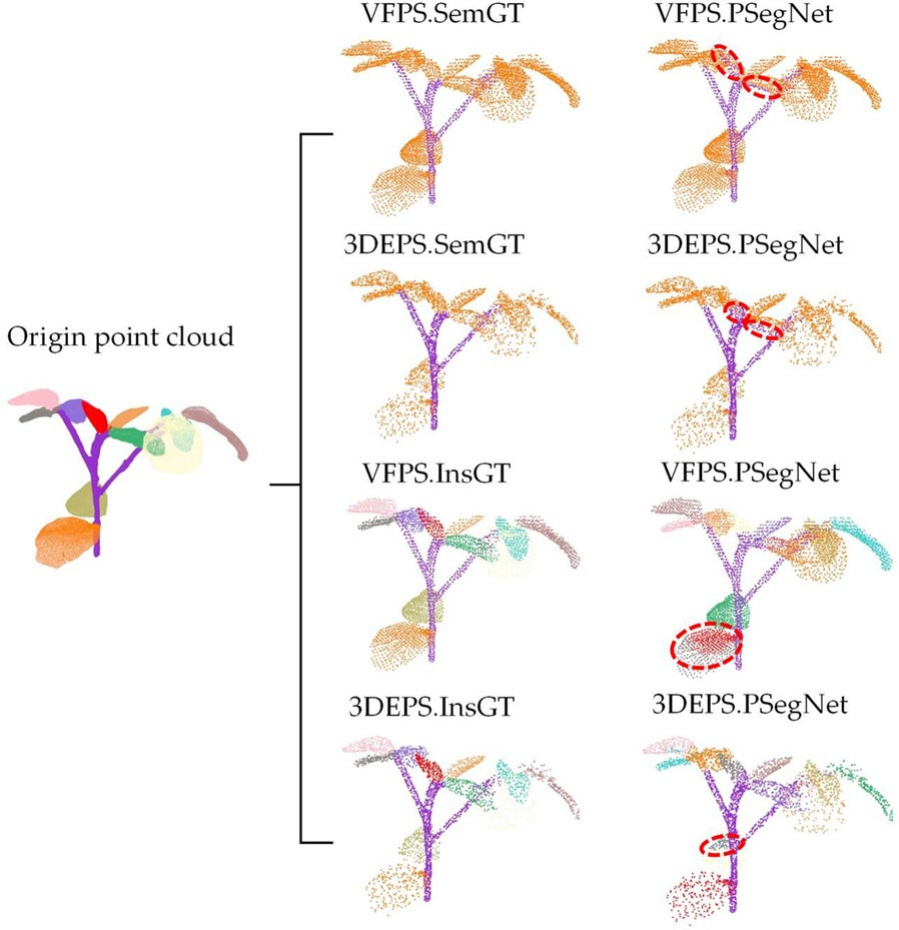
Comparison of the best and worst down-sampling strategies for qualitative segmentation results on the PSegNet for Soybean-MVS dataset. Each point cloud segmented by PSegNet is contrasted with its corresponding Ground Truth (SemGT or InsGT), and evident segmentation errors are highlighted by dotted red circles for better visualization. Please note that for the instance segmentation, different colors are only used to distinguish between different adjacent leaf organ instances, and there is no correspondence between leaf color and leaf index

Table 7 shows the quantitative semantic segmentation results on the PointNet +  + network under five down-sampling strategies for Soybean-MVS dataset. The best result of each metric is highlighted in bold, and the second-best is underlined. Table 8 shows the quantitative semantic segmentation results on the DGCNN network under five down-sampling strategies. Table 9 shows the quantitative segmentation results on the ASIS network under five down-sampling strategies. Table 10 shows the quantitative segmentation results on the PlantNet under five down-sampling strategies. Table 11 shows the quantitative segmentation results on the PSegNet under five down-sampling strategies. From the above tables, it can be seen that for the Soybean-MVS dataset, 3DEPS obtains the best quantitative results on 3 networks out of 5 in total; RS and FPS also perform well on multiple networks. In order to reduce redundancy in visualization, we choose one representative network (PointNet + +) on semantic segmentation task and one representative network (PSegNet) on instance segmentation task to show qualitative results. The PointNet +  + semantic segmentation results of UVS (the best strategy on PointNet + +) and 3DEPS (the worst strategy) are compared in Fig. 12, in which the UVS result has fewer errors than the 3DEPS counterpart. The PSegNet segmentation results on 3DEPS (the best strategy) data and VFPS (the worst strategy) data are contrasted in Fig. 13, 3 DEPS has fewer errors.

**Table 7:** Quantitative comparison of the semantic segmentation performance across five down-sampling strategies on PointNet +  + for the Soybean-MVS dataset

PointNet + +	Semantic Segmentation (%)				Score	AveDiff (%)
	Precision	Recall	F1	IoU		
FPS	72.42	70.18	71.33	58.91	11	1.91
RS	**73.82**	69.85	70.31	42.63	6	5.97
UVS	73.11	**73.27**	**72.98**	**60.44**	**17**	**0.17**
VFPS	71.19	68.66	69.56	57.42	2	3.42
3DEPS	70.04	66.63	67.62	53.26	0	5.74

**Table 8:** Quantitative comparison of the semantic segmentation performance across five down-sampling strategies on DGCNN for the Soybean-MVS dataset

DGCNN	Semantic Segmentation (%)				Score	AveDiff (%)
	Precision	Recall	F1	IoU		
FPS	90.93	88.58	89.68	82.00	0	2.73
RS	91.51	91.68	91.59	84.62	12	0.68
UVS	90.95	89.03	89.93	82.36	5	2.46
VFPS	91.08	86.93	88.83	80.77	1	3.62
3DEPS	**92.13**	**92.20**	**92.16**	**85.63**	**18**	**0.00**

**Table 9:** Quantitative comparison of both semantic segmentation and instance segmentation performances across five down-sampling strategies on the ASIS network for the Soybean-MVS dataset

ASIS	Semantic Segmentation (%)				Instance Segmentation (%)				Score	AveDiff (%)
	**Precision**	**Recall**	**F1**	**IoU**	**Cov**	**WCov**	**mPrec**	**mRec**		
FPS	**91.47**	85.05	87.81	79.27	28.83	**35.12**	**37.79**	10.69	**27**	**1.45**
RS	89.66	85.27	86.95	77.32	**33.07**	33.25	35.74	**11.72**	15	1.83
UVS	89.30	85.04	86.94	77.97	28.22	33.85	37.21	10.29	6	2.35
VFPS	88.36	80.29	83.53	73.49	27.02	34.06	36.64	10.38	6	4.23
3DEPS	89.32	**88.83**	**89.06**	**80.60**	28.52	29.34	31.72	09.06	18	2.65

**Table 10:** Quantitative comparison of both semantic segmentation and instance segmentation performances across five down-sampling strategies on the PlantNet network for the Soybean-MVS dataset

PlantNet	Semantic Segmentation (%)				Instance Segmentation (%)				Score	AveDiff (%)
	**Precision**	**Recall**	**F1**	**IoU**	**Cov**	**WCov**	**mPrec**	**mRec**		
FPS	86.79	87.30	87.03	78.04	45.06	**51.52**	**51.27**	**27.08**	19	**1.37**
RS	88.95	88.89	88.92	80.27	**45.54**	45.75	46.32	24.70	19	1.97
UVS	86.23	86.87	86.54	77.31	43.56	50.93	49.82	25.03	8	2.35
VFPS	86.80	86.55	86.69	77.61	38.95	46.35	45.41	19.89	3	4.60
3DEPS	**89.61**	**89.36**	**89.48**	**81.26**	45.37	46.21	44.36	24.70	**23**	1.84

**Table 11:** Quantitative comparison of both semantic segmentation and instance segmentation performances across five down-sampling strategies on the PSegNet network for the Soybean-MVS dataset

PSegNet	Semantic Segmentation (%)				Instance Segmentation (%)				Score	AveDiff (%)
	**Precision**	**Recall**	**F1**	**IoU**	**Cov**	**WCov**	**mPrec**	**mRec**		
FPS	86.75	86.76	86.75	77.65	45.40	**51.19**	48.76	26.65	15	2.11
RS	**89.13**	**89.01**	**89.06**	**80.50**	46.40	46.54	47.33	27.31	24	1.43
UVS	85.82	86.81	86.30	76.96	44.01	50.93	49.17	25.14	7	2.70
VFPS	85.13	86.16	85.61	76.08	38.68	45.52	43.67	20.10	0	5.73
3DEPS	88.64	88.31	88.47	79.67	**47.99**	48.96	**50.27**	**29.64**	**26**	**0.60**

Though 3DEPS tends to obtain the best result on evaluated networks, it is still not assertive that 3DEPS is the most suitable down-sampling strategy for high-precision plant models. Soybean-MVS is a single-species dataset that contains only 102 samples, and its only two semantic classes in training could result in overfitting, which further causes instability in learning. Given a network learned with increasing instability and decreasing generality, even 3DEPS did obtain the best on Soybean-MVS, there is no firm guarantee that 3DEPS can still obtain the best on future high-precision multi-species dataset. In addition, the strategies 3DEPS, UVS, and VFPS contain multiple steps on which manual parameter tuning and operation are needed, and the values of parameters are different from the case of the previous dataset. On the previous dataset, the algorithmic time costs of five strategies have no big differences; all of them running on real time or quasi-real time. However, on the Soybean-MVS dataset, the differences in speed start to appear. Considering the algorithmic time in automated processing only, the time cost comparison result is RS < VFPS≈UVS < 3DEPS < FPS; but if adding manual processing time into consideration, the time comparison result is RS < FPS < 3DEPS < VFPS≈UVS. The fact that FPS strategy becomes the slowest in pure algorithmic time cost comparison is not hard to imagine, as its complexity $O(Mn)$ quickly becomes formidable on a Soybean-MVS sample with more than 60,000,000 points. The data augmentation on FPS for the total training dataset even costs hours. Therefore, in practical FPS sampling on high-precision dataset, we recommend to first sample a very dense point cloud with RS to a scale of less than 1, 000,000 points, and then conduct FPS. The high time costs of UVS and VFPS are due to their voxelization tuning tests; fixing the suitable voxel size costs a lot of time, even running automatically by well-programed code. 3DEPS comprises a boundary detection program that needs parameter tuning, and two separate rounds of FPSs on smaller point sets; therefore, 3DEPS has restricted efficiency on high-precision datasets.

## Conclution

Currently, most deep networks for 3D plant phenotyping have only tested a single down-sampling algorithm such as FPS to prepare training sets and test sets. As far as we know, this research is the first comprehensive study of the cross relationship between multiple down-sampling strategies and the performances of popular networks for plant point clouds. The experiments show that there is currently no strict golden rule on down-sampling strategy for deep learning of crop point clouds; however, we have still summarized several suggestions on how to select a most suitable down-sampling strategy. First, for the networks that only carry out the semantic segmentation task, 3DEPS and UVS are easy to obtain satisfactory segmentation results. Second, on complex dual-function point cloud segmentation networks, 3DEPS, UVS, and VFPS usually generate satisfactory segmentation performance. Third, by comparing the differences on quantitative segmentation metrics, we have found out that the 3DEPS working under the optimal parameters is the most stable down-sampling strategy in experiments. Even if 3DEPS is not the best, it usually has very close performance metrics against the top performer.

In future, we are going to focus on the design of new down-sampling strategies that compute efficiently and perform effectively on advanced networks. Moreover, we are also trying to increase the diversity of species and the number of samples in our plant point cloud dataset.

## Data Availability

The dataset and code associated with this study will be available upon request.
